# Transcriptome Analyses of the Honeybee Response to *Nosema ceranae* and Insecticides

**DOI:** 10.1371/journal.pone.0091686

**Published:** 2014-03-19

**Authors:** Julie Aufauvre, Barbara Misme-Aucouturier, Bernard Viguès, Catherine Texier, Frédéric Delbac, Nicolas Blot

**Affiliations:** 1 Clermont Université, Université Blaise Pascal, Laboratoire “Microorganismes: Génome et Environnement”, BP 10448, Clermont-Ferrand, France; 2 CNRS, UMR 6023, LMGE, Aubière, France; Ghent University, Belgium

## Abstract

Honeybees (*Apis mellifera*) are constantly exposed to a wide variety of environmental stressors such as parasites and pesticides. Among them, *Nosema ceranae* and neurotoxic insecticides might act in combination and lead to a higher honeybee mortality. We investigated the molecular response of honeybees exposed to *N. ceranae*, to insecticides (fipronil or imidacloprid), and to a combination of both stressors. Midgut transcriptional changes induced by these stressors were measured in two independent experiments combining a global RNA-Seq transcriptomic approach with the screening of the expression of selected genes by quantitative RT-PCR. Although *N. ceranae*-insecticide combinations induced a significant increase in honeybee mortality, we observed that they did not lead to a synergistic effect. According to gene expression profiles, chronic exposure to insecticides had no significant impact on detoxifying genes but repressed the expression of immunity-related genes. Honeybees treated with *N. ceranae*, alone or in combination with an insecticide, showed a strong alteration of midgut immunity together with modifications affecting cuticle coatings and trehalose metabolism. An increasing impact of treatments on gene expression profiles with time was identified suggesting an absence of stress recovery which could be linked to the higher mortality rates observed.

## Introduction

Honeybees (*Apis mellifera*), like any living organism, are constantly exposed to a wide variety of biotic and abiotic stressors. Among those, pathogens and pesticides represent important variables influencing honeybee survival [1]–[4]. The microsporidian parasite *Nosema ceranae*, one of the most common pathogens of the honeybee, is a unicellular eukaryote and invasive intracellular parasite infecting *A. mellifera* midgut and inducing a disease named nosemosis [5]. This worldwide emerging parasite presents a high prevalence in honeybee colonies [6], [7]. *N. ceranae* infection has been shown to affect the honeybee health through immune suppression [8]–[10], energetic stress [11]–[13], degeneration of gut epithelial cells [10], [14] and reduction of lifespan [10], [15], [16] and therefore might lead to colony depopulation [15].

Honeybees are also frequently exposed to neonicotinoid and phenylpyrazole insecticides which are neurotoxic compounds intensively used on crops worldwide against arthropod pests [17], [18]. Inside their colonies, honeybees can be orally or topically exposed to these insecticides as diverse matrices (*i.e.* pollen, honey, wax) can be contaminated with low concentrations of these compounds [18]–[21]. Nonetheless, chronic exposure to low doses of neonicotinoids and phenylpyrazoles can have sublethal effects on honeybee [22], [23] such as impairment on cognition [24]–[26] and flight behaviour [27]–[31]. Moreover, low doses of the phenylpyrazole fipronil or the neonicotinoid imidacloprid can lead to a significant decrease in honeybee survival following chronic exposure [32]–[34].

Environmental stressors might interact with each other and potentiate their effects on organisms’ health and survival [35], [36]. Interactions between stressors in honeybees may be partly responsible for the severe colony losses recorded worldwide for more than ten years [1]–[4]. *N. ceranae* and insecticides were shown to act synergistically on the reduction of the honeybee lifespan. Synergistic interaction is defined as a combination of stressors that results in a greater effect than expected from cumulative independent exposures [35]. A synergistic effect on mortality was observed in honeybees co-exposed to *Nosema* spp. spores and imidacloprid [37]. *N. ceranae* and fipronil combinations also led to a synergistic effect on the honeybee mortality, whatever the sequence of exposure to stressors [38], [39].

Only few data have been collected regarding the molecular honeybee response to *N. ceranae* and insecticides and none to their combination. In insects, the immune and detoxification systems respond quickly to chemical and biological stresses [40] and are well expressed in the gut [41], [42] given that this organ is the site of exposure to many stressors. In honeybee, the midgut is the site of infection by *N. ceranae* but also the main site of exposure to orally administered chemicals. Our objective was to investigate the honeybee response to biotic and abiotic environmental stressors by measuring the midgut transcriptional changes induced by the parasite *N. ceranae* and one neurotoxic insecticide (fipronil or imidacloprid), alone or in combination. For this purpose, we performed two independent experiments combining a global RNA-Seq transcriptomic approach (Exp. 1) with the screening of the expression of selected genes by quantitative RT-PCR (Exp. 2). The global RNA-Seq approach allowed the identification of several genes of interest which were further analysed by quantitative RT-PCR in Exp. 2, together with genes chosen from the available literature.

## Materials and Methods

### Bees, Parasites and Insecticides

Experiments 1 and 2 were performed in September 2012 and April 2013 respectively, with *Apis mellifera* emerging honeybees taken from different colonies of the same apiary at the Laboratoire Microorganismes : Génome et Environnement (UMR 6023, Université Blaise Pascal, Clermont-Ferrand, France). Frames of sealed brood were placed in an incubator in the dark at 33°C under humidified atmosphere. Emerging honeybees were collected and distributed into different experimental groups of 165 and 140 individuals for Exp. 1 and Exp. 2 respectively and placed in cages. In order to mimic the colony environment, a 5 mm piece of Beeboost (Phero Tech Inc.) releasing 5 queen’s mandibular pheromones was placed in each cage. During all the experiment, honeybees were fed *ad libitum* with 50% (w/v) sugar syrup supplemented with 1% (w/v) Provita’Bee (Vetopharm Pro). Every day, feeders were replaced, dead bees were counted and removed, and the sucrose consumption was quantified. Bees were either not treated (control), infected with *N. ceranae*, chronically exposed to low concentrations of an insecticide (fipronil or imidacloprid), or exposed to both *N. ceranae* and insecticide (fipronil or imidacloprid).

*N. ceranae* spores were obtained according to Vidau *et al.* (2011) [38]. The spore concentration was determined by counting using a haemocytometer chamber. *N. ceranae* species was confirmed by PCR according to Martín-Hernández *et al.* (2007) [43]. Emerging honeybees were individually infected by feeding with 125,000 spores of *N. ceranae* in 3 μL of 50% sucrose solution using a micropipette. Control honeybees were treated with a sucrose solution devoid of *N. ceranae* spores.

Stock solutions of fipronil (1 or 2 g/L for Exp. 1 and Exp. 2 respectively) and imidacloprid (2 g/L) were prepared in DMSO and diluted in sucrose to a final concentration of 1.3 μg/L (Exp. 1) or 2 μg/L (Exp. 2) for fipronil and 2 μg/L for imidacloprid with 0.1% DMSO (v/v). Emerging honeybees were exposed *ad libitum* to the contaminated feeding syrup for 7 days. The insecticide consumption was quantified by measuring the daily amount of contaminated syrup consumed per cage reported per living honeybee. Control honeybees were fed *ad libitum* with 0.1% DMSO-containing sugar syrup.

### RNA Extraction

Honeybee midguts were dissected on ice, pooled and immediately homogenized in 400 μL TRIzol Reagent (Life Technologies) using first a microtube pestel and then a BioSpec Mini-BeadBeater (3 pulses for 2 min at 30 Hz) after addition of 150 mg of 0.5 mm glass beads. Following phase separation according to the manufacturer, the aqueous phase was supplemented with 0.015 volume of β-mercaptoethanol and 1 volume of 70% ethanol and transferred to an RNeasy spin column (RNeasy Mini Kit, Qiagen). Total RNA was isolated according to the kit instructions. Genomic DNA was removed using the RNA-free DNase set (Qiagen) twice during the RNA extraction. RNA was quantified by spectrophotometry using the ND-1000 (Nanodrop).

### RNA-Seq Analysis of Differentially Expressed Genes

Two RNA samples were extracted for each experimental group from pools of 3 midguts. RNA quality and concentration were checked using a RNA 6000 Nano Chip on the 2100 Bioanalyzer (Agilent Technologies). Six micrograms of each RNA sample were provided to Montpellier GenomiX (MGX, Institut de Génomique Fonctionnelle, Montpellier, France) for an additional RNA quality control and for library preparation and sequencing (50 bp single-end reads) on an HiSeq 2000 (Illumina) following the manufacturer’s protocol. Image analyses and base-calling were conducting using the HiSeq Control Software 1.4.5.0 and RTA component 1.12.4.0 (Illumina). Sequences were mapped on the *Apis mellifera* genome (version Amel_4.5 downloaded from NCBI), allowing up to two mismatches in alignments, and tags that matched predicted transcripts were counted using CASAVA 1.8.2 (Illumina).

The R package DESeq was used to normalize data and determine which genes were differentially expressed among treatments [44]. Genes were considered to be differentially expressed between two treatments at an adjusted p-value <0.1. The p-values were adjusted for multiple testing with the Benjamini-Hochberg procedure which controls for false discovery rate. A principal component analysis (PCA) was performed under PAST [45] to evaluate the samples distribution according to their expression profiles. This analysis was performed using the log of normalized data corresponding to the 3001 genes showing an adjusted p-value <0.1 in at least one pairwise comparison.

### Quantitative Real-time PCR

cDNA synthesis was performed in a 20 μL reaction containing 0.5 μg of total RNA, 0.5 μg of oligo(dT)_12–18_ primers (Life Technologies), 0.5 mM of each dNTP and 200 U of SuperScript III Reverse Transcriptase (Life Technologies).

All real-time quantitative PCR analyses were performed in 96-well plates on a Mastercycler ep Realplex^2^ thermocycler (Eppendorf) monitored by the Realplex software (version 1.5) using the primers and conditions listed in Table S1. All primer sets, whether they were already published or newly designed, have been validated according to their specificity, linearity, efficiency and amplification reproductibility (Table S1). qPCR reaction mixtures consisted in 5 μL of 1∶16 diluted cDNA, the appropriate concentration of each primer and 10 μL of 2X Absolute Blue QPCR SYBR Green Mix (Thermo Scientific) in a final volume of 20 μL. Negative controls (without cDNA) were included in each reaction set. The PCR program consisted in an initial step at 95°C for 15 min, and 40 cycles of 95°C for 15 s, specific annealing temperature for 30 s and 72°C for 30 s. Fluorescence was measured in each cycle after the elongation step. The specificity of the reaction was checked by analysing the melting curve of the final amplified product. The amplification results were expressed as the threshold cycle number (C_T_), which represents the number of cycles needed to generate a fluorescent signal greater than a predefined threshold. C_T_ values were normalized by subtracting the C_T_ value of the RpS5a reference gene from the corresponding cDNA sample. For every gene, data were analysed under PAST to determine if they fitted a normal distribution. The variation in gene transcript levels between different groups was evaluated by two-way ANOVA followed by a Student’s t-test. When data did not fit a normal distribution, the non-parametric Kruskal-Wallis test was applied. p-values below 0.05 were considered significant.

## Results

### Whole Honeybee Transcriptome Response to *N. ceranae* and/or Fipronil using RNA-Sequencing (Exp. 1)

#### Survival analysis

**Figure 1:**
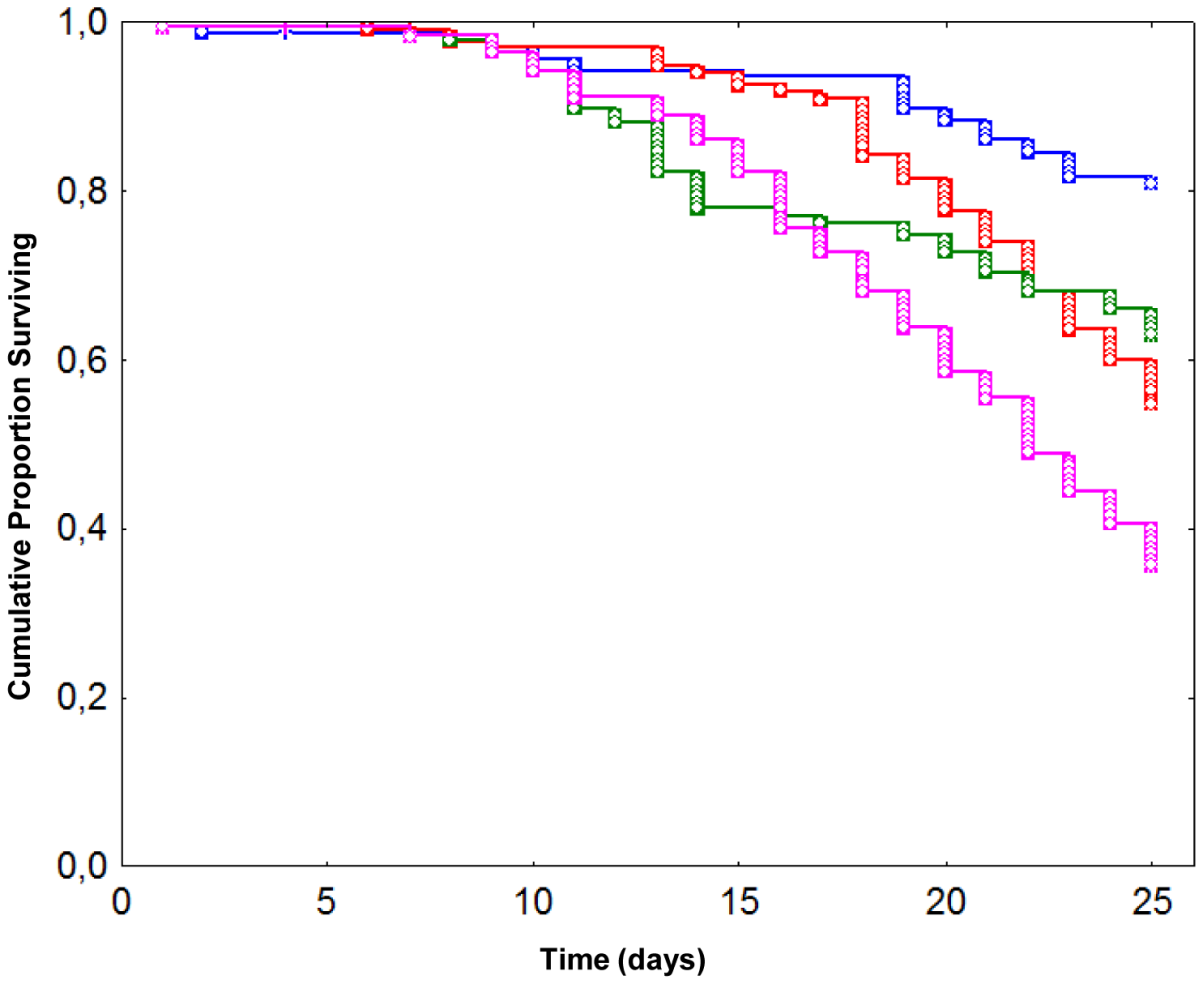
Effect of *N. ceranae* and fipronil, alone or in combination, on honeybee survival. Data give the cumulative proportion of surviving honeybees exposed to no treatment (blue), *N. ceranae* (red), fipronil (green), or a *N. ceranae*-fipronil combination (pink). *N. ceranae*-treated honeybees were individually infected at their emergence (day 0) and fipronil-treated ones were chronically and orally exposed to fipronil (1.3 μg/L) from day 0 to day 7. Data from 165 honeybees per experimental condition were analysed using the Kaplan-Meier method.

In this experiment, newly emerged honeybees were exposed to (i) no treatment, (ii) *N. ceranae* (125,000 spores/bee), (iii) chronic exposure to fipronil (1.3 μg/L for 7 days) or (iv) the combination of both stressors. Survival analysis revealed that each treatment, applied alone or in combination, led to a significant decrease (p≤0.05, one-tailed χ^2^ test) in honeybee survival compared to control (Figure 1). As expected, control honeybees presented the lowest mortality rate (19%) at the end of the experiment, *i.e.* 25 days after emergence. Mortality rates were significantly increased in honeybees exposed to *N. ceranae* (45%, p<0.001) or fipronil (37%, p<0.001) alone compared to control. Both factors, when applied alone, had a similar impact on honeybee survival (p = 0.070). The mortality rate of honeybees co-exposed to both factors reached a maximum of 64%, and was significantly different from other experimental groups (p<0.001). However, while the *N. ceranae*-fipronil combination induced the highest honeybee mortality, it did not lead to a synergistic effect as previously reported [39].

As expected from our previous study [39], *N. ceranae* did not affect host sucrose consumption, nor did insecticide exposure affect parasite development (data not shown). Honeybees exposed to fipronil and to the *N. ceranae*-fipronil combination absorbed a similar daily quantity of fipronil of 1/209^th^ (19.9±5.1 pg/day/bee) and 1/201^th^ of the LD50 (20.7±6.9 pg/day/bee) respectively (LD50 of 4.17 ng/bee [46]). Although these doses were much lower than the molecule LD50, the fipronil treatment could not be considered as sublethal in this study as it induced a significant increase in honeybee mortality.

#### Identification of differentially expressed genes

The midgut transcriptome modifications induced by parasitism and exposition to insecticide, acting alone or in combination, were determined 1 and 7 days after the treatments’ initiation by whole transcriptome sequencing (RNA-Seq). A total of 16 RNA-Seq libraries were generated with 2 libraries for each experimental group. The number of sequence reads that mapped the honeybee genome varied between 10,000,000 and 27,000,000 per library, except for one replicate collected at day 7 in the group of honeybees co-exposed to *N. ceranae* and fipronil where only 14.2% of the reads (3,961,114 reads) matched the genome. This sample contained a high amount of *Varroa destructor* virus sequences and was discarded from further analyses. Overall, 10,061 honeybee genes have been detected in either library (Table S2). The R package DESeq [44] was used to normalize data and perform all possible pairwise comparisons to determine the differentially expressed genes between experimental groups. In order to validate the RNA-Seq data, 8 genes showing differential expression in at least two pairwise comparisons were selected. qRT-PCR assays on these genes confirmed both the direction and the magnitude of changes (Spearman rank correlation ρ = 0.722, n = 72, p<0.001) (Figure S1).

**Figure 2:**
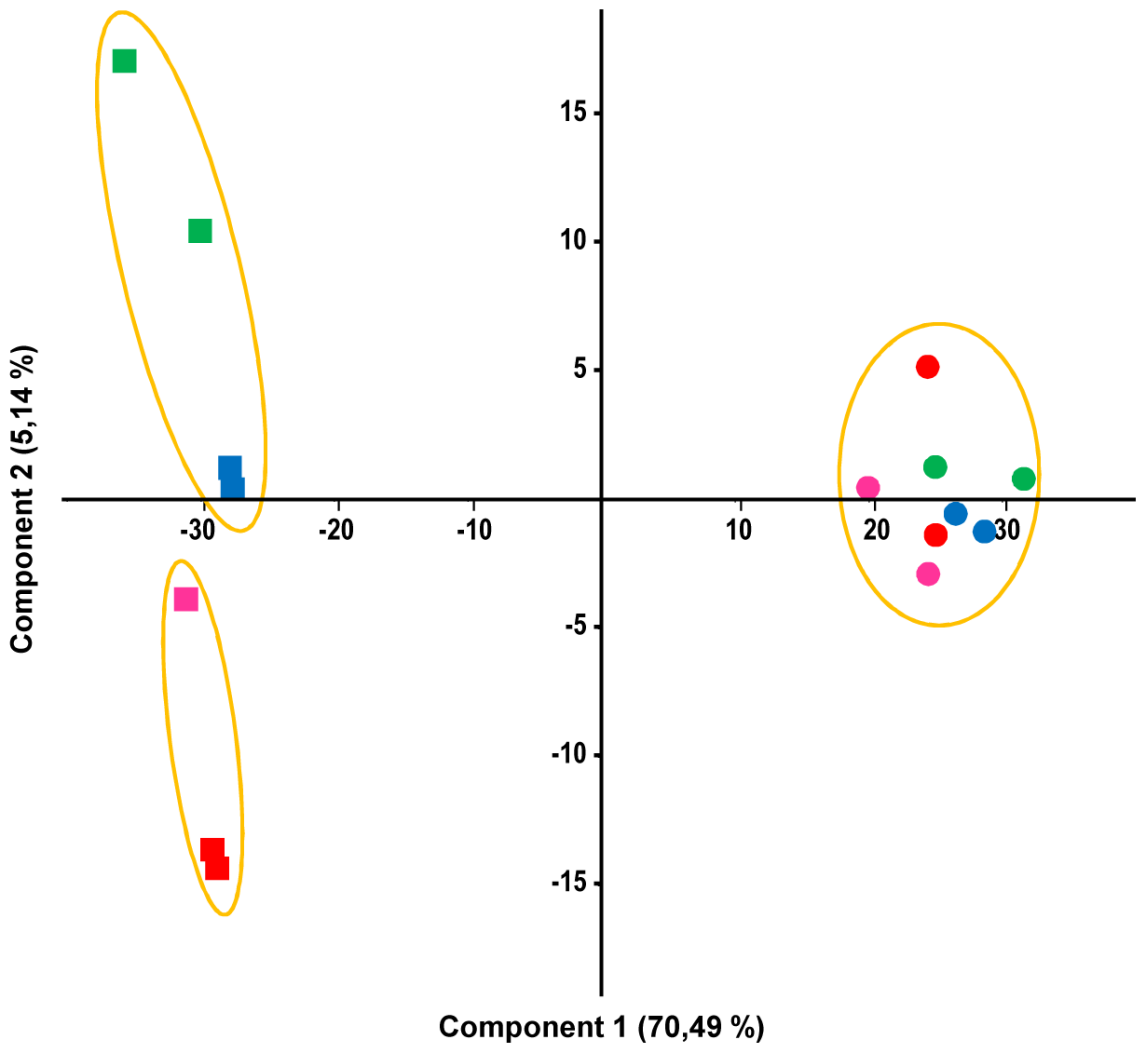
Principal component analysis of RNA-Seq data. Gene expression changes were investigated at day 1 (circles) or 7 (squares) in honeybees exposed to no treatment (blue), *N. ceranae* (red), fipronil (green), or a *N. ceranae*-fipronil combination (pink). The PCA was performed using normalized RNA-Seq data of a set of 3001 genes showing an adjusted p-value <0.1 in at least one pairwise comparison.

A PCA was performed on normalized data corresponding to a set of 3001 genes showing an adjusted p-value <0.1 in at least one pairwise comparison in the whole data (Figure 2). The first (70.5%) and second (5.1%) components represent most of the differential expression pattern with a cumulative proportion of 75.6%. This analysis revealed a clear segregation between samples collected at day 1 and those collected at day 7. The PCA plot also showed that samples collected at day 1 were all gathered together, while those collected at day 7 were segregated in a treatment-dependent way, possibly more strongly upon parasite infection. Therefore, honeybee ageing might have highly influenced gene expression in our experiment but exposure to parasite and/or insecticide might also have influenced it 7 days after treatment initiation. Indeed, pairwise comparisons performed on samples collected on day 1 only resulted in 17 genes whose expression was significantly modified between two experimental groups (*i.e.* showing at least one adjusted p-value <0.1 among all pairwise comparisons), while expression profiles were more affected on day 7 with 104 genes whose expression was affected.

#### Honeybee genes responding to *Nosema* infection and fipronil intoxication

In order to determine which genes were involved in honeybee response to *N. ceranae* and/or exposure to fipronil, we focused on pairwise comparisons performed between treated groups and controls at the same day. Overall, we found 71 genes whose expression is significantly modified in response to at least one of the treatments 1 or 7 days after the experiment initiation (Table 1). The honeybee response to treatments is particularly low 1 day after treatment initiation, with only 10 genes whose expression is significantly modified, compared to the 63 genes detected from data collected on day 7.

**Table 1:** Honeybee genes whose expression is significantly modified in response to at least one treatment (*N. ceranae* infection, chronic exposure to fipronil or a combination of both stressors) compared to the untreated control, 1 or 7 days after the experiment initiation.

Locus (Gene)	Product	*N. ceranae*vs. control	Fipronilvs. control	*N. cer.*+ fip.vs. control
**1 day after treatment initiation**				
406065 (Wat)	worker-enriched antennal transcript	**2.32 (0.056)**	0.44 (1)	0.86 (1)
726421	membrane metallo-endopeptidase-like	**1.19 (0.051)**	0.47 (1)	0.96 (0.964)
408864	waprin-Phi1-like variant 2	**−0.84 (0.056)**	−0.13 (1)	−0.11 (1)
552195	–	**−1.32 (0.051)**	−0.34 (1)	−0.50 (1)
551454	voltage-dependent calcium channel subunit	**−1.45 (0.028)**	−0.01 (1)	−0.77 (1)
411602	dynein heavy chain 6, axonemal-like	**−1.54 (0.067)**	−1.21 (1)	−1.16 (1)
408365 (serp)	serpentine	**−1.56 (0.056)**	−0.58 (1)	−0.51 (1)
100576126	–	−0.81 (1)	−0.30 (1)	**−1.58 (0.090)**
100578599	–	**−2.93 (0.000)**	−0.63 (1)	−0.94 (1)
725041	slit homolog 2 protein-like	**−3.08 (0.008)**	−0.54 (1)	−1.92 (1)
**7 days after treatment initiation**				
724556 (CPR17)	cuticular protein 17	−0.38 (1)	1.57 (1)	**6.41 (0.000)**
408650	inositol oxygenase-like	−0.05 (1)	**4.54 (0.088)**	2.13 (1)
100578048	–	**2.37 (0.001)**	−0.24 (1)	**3.83 (0.000)**
725123	–	1.31 (1)	0.85 (1)	**3.57 (0.029)**
413894 (Y-e3)	yellow-e3	1.33 (1)	1.71 (1)	**3.44 (0.002)**
100576797	acyl-CoA Delta(11) desaturase-like	1.36 (1)	**2.79 (0.055)**	0.55 (1)
551600 (Cht5)	chitinase 5 variant 2	**2.61 (0.001)**	−0.12 (1)	**2.49 (0.011)**
412202 (CPR28)	cuticular protein 28	0.15 (1)	0.15 (1)	**2.35 (0.055)**
727287	–	−0.33 (1)	−0.14 (1)	**2.20 (0.000)**
727037	lipase member H-A-like	0.86 (1)	1.42 (0.372)	**2.11 (0.055)**
100578599	–	**2.07 (0.008)**	−0.48 (1)	1.78 (0.196)
408645	–	−0.02 (1)	0.32 (1)	**1.93 (0.008)**
100577518	leucine-rich repeats and immunoglobulin-like	**1.48 (0.005)**	0.22 (1)	0.56 (1)
552210 (Faa)	fumarylacetoacetase	**1.38 (0.006)**	0.41 (1)	1.31 (0.142)
725272	histone H3-like	**1.36 (0.000)**	0.29 (1)	0.88 (0.568)
412220	vesicular glutamate transporter 2-like	0.52 (1)	**0.87 (0.066)**	**1.28 (0.001)**
406132	histone H4	**1.26 (0.022)**	−0.01 (1)	0.73 (1)
551143 (Inos)	inositol-3-phosphate synthase	−0.13 (1)	**1.16 (0.012)**	0.11 (1)
413858	–	0.05 (1)	0.45 (1)	**0.97 (0.029)**
551924	–	**0.95 (0.007)**	0.14 (1)	0.55 (1)
408669	–	**0.95 (0.057)**	−0.16 (1)	0.40 (1)
413768	2-oxoglutarate dehydrogenase	0.18 (1)	0.30 (1)	**0.95 (0.078)**
552244	–	**0.85 (0.051)**	0.18 (1)	0.56 (1)
724336	tRNA dimethylallyltransferase	**0.83 (0.051)**	0.15 (1)	0.65 (1)
412282	leucyl-tRNA synthetase, cytoplasmic-like	**0.75 (0.083)**	−0.48 (1)	−0.01 (1)
409932	B(0,+)-type amino acid transporter 1-like	**0.68 (0.099)**	−0.02 (1)	0.47 (1)
410527	probable cation-transporting ATPase	**0.62 (0.088)**	−0.18 (1)	0.49 (1)
412843 (Pepck)	phosphoenolpyruvate carboxykinase	**−0.62 (0.081)**	−0.01 (1)	−0.27 (1)
725756	beta-galactosidase	**−0.75 (0.083)**	**−0.72 (0.066)**	−0.44 (1)
725777	LIM and SH3 domain protein Lasp-like	−0.61 (0.347)	**−0.75 (0.040)**	−0.58 (1)
726414	–	−0.13 (1)	**−0.76 (0.051)**	−0.46 (1)
725466 (Fur1)	furin-like protease 1	−0.29 (1)	**−0.77 (0.088)**	−0.23 (1)
726965	–	**−0.79 (0.016)**	−0.62 (0.222)	−0.82 (0.12)
410767 (Nedd9)	neural precursor cell expressed, developmentallydown-regulated gene 9	−0.31 (1)	**−0.82 (0.012)**	−0.55 (1)
725862	–	−0.13 (1)	**−0.84 (0.085)**	−0.74 (0.804)
413117	proton-coupled amino acid transporter	0.16 (1)	**−0.86 (0.010)**	−0.49 (1)
409185	–	−0.45 (1)	−**0.86 (0.072)**	−0.64 (1)
724832 (inx2)	innexin 2	−0.37 (1)	−**0.86 (0.006)**	−0.59 (1)
727092	POU domain, class 2, transcription factor 3-like	0.16 (1)	−**0.92 (0.012)**	−0.28 (1)
408664 (hipk)	homeodomain interacting protein kinase	−0.70 (0.123)	−0.59 (0.331)	−**0.94 (0.048)**
100578870	–	−0.48 (1)	−**0.97 (0.017)**	−0.26 (1)
100576695	–	−0.76 (0.631)	−**0.99 (0.095)**	−0.82 (1)
412007	facilitated trehalose transporter	−0.18 (1)	−0.24 (1)	−**1.00 (0.083)**
408358	–	−**1.06 (0.005)**	−**0.86 (0.051)**	−0.87 (0.327)
413575	facilitated trehalose transporter	−0.37 (0.926)	−**0.98 (0.000)**	−**1.06 (0.004)**
409628 (CDase)	neutral ceramidase	−**1.08 (0.003)**	−0.57 (0.958)	−1.04 (0.102)
725671	homeobox protein Nkx-2.5-like	−0.34 (1)	−**1.09 (0.001)**	−0.69 (1)
410658 (Lim3)	Lim3 homeobox	−**0.98 (0.027)**	−**1.11 (0.005)**	−**1.08 (0.087)**
726990	–	−0.62 (0.407)	0.03 (1)	−**1.18 (0.06)**
413242	heparan sulfate N-deacetylase	−**1.03 (0.001)**	−**0.83 (0.012)**	−**1.31 (0.000)**
100576640	–	−0.86 (0.106)	−**1.34 (0.000)**	−0.68 (1)
410484	trehalase	−**1.35 (0.000)**	−0.47 (0.922)	−**0.92 (0.032)**
100577390	fibroblast growth factor 18-like	−**1.33 (0.003)**	−0.85 (0.222)	−**1.39 (0.029)**
413645 (SP22)	serine protease 22	−0.57 (0.442)	−0.41 (1)	−**1.41 (0.001)**
410638 (Ddc)	dopa decarboxylase	−**1.42 (0.003)**	−0.54 (1)	−1.24 (0.102)
100576509	–	−0.20 (1)	−**1.44 (0.001)**	−0.76 (1)
411353	lipase 3-like	−**0.79 (0.006)**	−0.39 (1)	−**1.45 (0.000)**
406114	alpha-amylase	−**1.77 (0.000)**	−0.59 (1)	−0.65 (1)
409626 (SP40)	serine protease 40	−**1.18 (0.022)**	−0.28 (1)	−**1.96 (0.000)**
100576126	–	−**2.14 (0.006)**	−0.64 (1)	−**2.36 (0.029)**
726796	hydrocephalus-inducing protein homolog	−1.08 (0.436)	−0.53 (1)	−**3.20 (0.000)**
100578512	hydrocephalus-inducing protein-like	−**1.66 (0.049)**	−1.09 (0.691)	−**3.42 (0.000)**
100578545	–	−**2.13 (0.002)**	−0.65 (1)	−**4.38 (0.000)**

The log_2_ ratio of the normalized transcript content relative to the control at the same day is given together with the adjusted p-value in parentheses. Significant expression changes (adjusted p-value <0.1) are shown in bold.

The infection by *N. ceranae* induced various gene expression changes on day 7 compared to control (Table 1). *N. ceranae* led to a significant overexpression of the genes encoding the histones H3-like (Gene ID 725272) and H4 (Gene ID 406132). The expression levels of the serine proteases encoding genes SP40 (Gene ID 409626) and SP22 (Gene ID 413645) were significantly decreased in the *N. ceranae* and *N. ceranae*-fipronil treatments for the former, and to the *N. ceranae*-fipronil treatment only for the latter. Several genes involved in chitin metabolism and cuticle coatings, that constitute an important barrier defence in honeybees, were activated in experimental groups: the chitinase 5 encoding gene (Gene ID 551600) in response to *N. ceranae* and *N. ceranae*-fipronil combination, and the genes encoding the cuticular proteins 17 (Gene ID 724556) and 28 (Gene ID 412202) in response to the *N. ceranae*-fipronil combination only. Additionally, parasitism decreased the expression of genes related to carbohydrates metabolism. The expression of the genes encoding the phosphoenolpyruvate carboxykinase (Gene ID 412843) and the facilitated trehalose transporter Tret1-like (Gene ID 412007) was reduced in bees exposed to *N. ceranae* and to the *N. ceranae*-fipronil combination respectively. Some treatments affected the expression of genes involved in amino acid metabolism and transport. The transcript amounts were higher for the B(0,+)-type amino acid transporter 1-like (Gene ID 409932) and the fumarylacetoacetase (Gene ID 552210) encoding genes in response to *N. ceranae*, and for the vesicular glutamate transporter 2-like encoding gene (Gene ID 412220) in response to fipronil and to *N. ceranae*-fipronil. In contrast, an inhibition of expression was observed for the dopa decarboxylase encoding gene (Gene ID 410638) in response to *N. ceranae*, and for the proton-coupled amino acid transporter 4-like encoding gene (Gene ID 413117) in response to fipronil alone. Fipronil also led to the overexpression of two genes related to carbohydrate metabolism, encoding an inositol-3-phosphate synthase (Gene ID 551143) and an inositol oxygenase-like (Gene ID 408650). Honeybees exposed to fipronil alone also showed a significant decrease in the expression of two genes encoding transcriptional factors: the homeobox protein Nkx-2.5-like (Gene ID 725671) and the POU domain, class 2, transcription factor 3-like (Gene ID 727092).

### Transcriptomic Profile of Selected Genes in Response to *N. ceranae*, Fipronil and Imidacloprid (Exp. 2)

#### Survival analysis

In this experiment, newly emerged honeybees were exposed to (i) no treatment, (ii) *N. ceranae* (125,000 sp/bee), (iii) chronic exposure to fipronil (2 μg/L), (iv) chronic exposure to imidacloprid (2 μg/L), (v) *N. ceranae*-fipronil combination or (vi) *N. ceranae*-imidacloprid combination. Survival analysis showed a general pattern which is quite similar to the one observed in Exp. 1 (Fig. S2). Briefly, all parasite and/or insecticide treatments, except for the imidacloprid treatment (p = 0.079), led to a significant decrease (p≤0.001) in honeybee survival compared to control. Moreover, mortality rates induced by *N. ceranae*-fipronil and *N. ceranae*-imidacloprid combinations were the highest (69% and 70% respectively after 12 days) and were significantly (p≤0.001) higher than the parasite or insecticide treatments applied alone. However, neither *N. ceranae*-fipronil nor *N. ceranae*-imidacloprid combination led to a synergistic effect on host mortality.

Regarding exposure to fipronil, honeybees treated with fipronil alone (46.1±17.7 ng/day/bee, LD50/90) and honeybees exposed to the *N. ceranae*-fipronil combination (43.1±12.2 ng/day/bee, LD50/97) consumed similar daily quantities of insecticide. Like in Exp. 1, fipronil doses received by honeybees in both experimental groups cannot be considered as sublethal. Honeybees exposed to imidacloprid also consumed similar daily quantities of insecticide, whether they were infected by *N. ceranae* (42.2±13.5 ng/day/bee, LD50/664) or not (45.1±17.6 ng/day/bee, LD50/621). As the mortality rate of honeybees treated with imidacloprid alone was not significantly different from the one measured in control bees, the imidacloprid dose received by individuals could be considered as sublethal.

#### Determination of differentially expressed genes by quantitative RT-PCR

Twenty four honeybee genes have been selected as potential expression markers for parasitism or exposure to insecticide. Among those, sixteen genes are involved in functions, such as immunity, detoxification and antioxidant reactions, that are activated in response to environmental stressors in honeybees. As an example, it has been shown that the expression of the genes encoding the hymenoptaecin and defensin 1 antimicrobial peptides (Gene ID 406142 and 406143) and the glucose dehydrogenase (Gene ID 551044) is significantly decreased several days after *N. ceranae* infection [8], [9]. Six genes showing differential expression in our RNA-Seq experiment have also been selected. The transcript levels of the 24 selected genes were determined for each experimental condition from four pools of five midguts 7 and 11 days after treatment initiation. Six and eight genes were significantly differentially expressed in at least one experimental group compared to control in samples collected at days 7 and 11 respectively (Table 2).

**Table 2:** Expression levels of 24 honeybee genes in response to various stressors, alone or in combination, 7 or 11 days after experiment initiation (d.a.i).

Locus (Gene)	Product	*N. ceranae vs.* control		Fipronil *vs.* control		Imidacloprid *vs.* control		*N. cer.*+ fip. *vs.* control		*N. cer.*+ imi. *vs.* control	
		7 d.a.i	11 d.a.i	7 d.a.i	11 d.a.i	7 d.a.i	11 d.a.i	7 d.a.i	11 d.a.i	7 d.a.i	11 d.a.i
725110	lysozyme 1	0.22 (0.517)	−2.12 (0.052)	−0.39 (0.463)	−**2.76 (0.007)**	0.06 (0.903)	−**2.70 (0.011)**	−0.12 (0.597)	−**3.10 (0.008)**	0.13 (0.783)	−2.39 (0.454)
522304	glutathione S-transferase S1	−0.01 (0.990)	−1.74 (0.442)	−0.22 (0.730)	−0.25 (0.443)	−0.31 (0.744)	0.04 (0.324)	−0.31 (0.625)	−2.42 (0.073)	−0.15 (0.842)	−2.26 (0.416)
100578512	hydrocephalus-inducing protein-like	−0.35 (0.885)	−0.05 (0.312)	−0.70 (0.194)	0.56 (0.312)	0.45 (0.309)	1.30 (0.312)	−0.63 (0.194)	−0.17 (0.817)	−0.10 (0.885)	−0.42 (0.312)
727092	POU domain, class 2, transcription factor 3-like	0.50 (0.211)	−1.37 (0.902)	−0.22 (0.545)	−0.41 (0.866)	−0.32 (0.451)	−0.50 (0.978)	−0.13 (0.744)	−2.44 (0.414)	0.37 (0.510)	−3.36 (0.350)
410747	GMC oxidoreductase 3	−**3.75 (0.030)**	−5.77 (0.061)	−**0.52 (0.030)**	−5.93 (0.061)	0.04 (0.885)	−3.96 (0.194)	−**2.63 (0.030)**	−6.65 (0.105)	−**2.56 (0.030)**	−**8.94 (0.030)**
410658	Lim3 homeobox	−**0.62 (0.000)**	−**4.17 (0.039)**	−**0.08 (0.000)**	−1.71 (0.548)	−**0.35 (0.000)**	−2.41 (0.249)	−**1.27 (0.000)**	−**7.15 (0.014)**	−**0.59 (0.000)**	−**6.06 (0.012)**
725154	serine protease 14	1.51 (0.061)	2.64 (0.061)	0.85 (0.112)	1.57 (0.112)	1.55 (0.061)	0.32 (0.885)	1.58 (0.061)	−0.24 (0.817)	1.77 (0.061)	0.61 (0.194)
409626	serine protease 40	−**2.80 (0.030)**	−3.43 (0.194)	−0.61 (0.147)	−0.34 (0.665)	−0.27 (0.665)	−1.00 (0.772)	−**2.55 (0.030)**	−3.90 (0.247)	−**1.88 (0.030)**	−**5.70 (0.030)**
551758	glucosinolate sulphatase	−0.83 (0.312)	−2.88 (0.194)	0.19 (0.665)	0.35 (0.665)	0.74 (0.312)	−0.59 (0.885)	−0.07 (1)	−0.58 (0.817)	0.17 (0.885)	−3.03 (0.471)
410484	trehalase	−0.76 (0.162)	−**4.61 (0.018)**	0.32 (0.408)	−0.48 (0.864)	0.81 (0.226)	−0.80 (0.935)	−**0.87 (0.043)**	−**5.15 (0.024)**	−0.07 (0.885)	−3.75 (0.091)
551600	chitinase 5	**2.85 (0.030)**	0.26 (0.312)	−0.07 (0.885)	−2.65 (0.312)	−0.10 (0.665)	−2.44 (0.194)	**2.13 (0.030)**	−1.55 (0.817)	**2.58 (0.030)**	−1.71 (0.471)
412150	endoplasmin-like	0.27 (0.662)	−0.05 (0.295)	−0.26 (0.479)	−0.38 (0.703)	−0.10 (0.905)	−0.52 (0.979)	−0.09 (0.874)	−1.95 (0.228)	0.65 (0.270)	−1.03 (0.361)
411758	catalase (1)	0.07 (0.885)	0.16 (0.216)	−0.11 (0.471)	−0.14 (0.885)	0.64 (0.665)	−0.22 (0.885)	0.20 (0.885)	−1.48 (0.817)	0.70 (0.312)	−1.25 (0.471)
443552	catalase (2)	−0.64 (0.391)	−**3.67 (0.046)**	−0.48 (0.520)	−1.19 (0.573)	−0.42 (0.611)	−1.56 (0.388)	−0.98 (0.189)	−2.61 (0.076)	−0.60 (0.465)	−3.59 (0.456)
406143	defensin 1	1.75 (0.112)	0.37 (0.471)	0.18 (0.885)	1.95 (0.471)	0.93 (0.665)	−0.45 (0.665)	1.50 (0.194)	3.14 (0.247)	1.41 (0.665)	2.43 (0.194)
406142	hymenoptaecin	−1.77 (0.323)	−**7.20 (0.008)**	−2.22 (0.286)	−**3.87 (0.037)**	−0.91 (0.607)	−3.63 (0.187)	−0.90 (0.600)	−5.15 (0.133)	−2.53 (0.184)	−**6.37 (0.023)**
100578995	Vanin-like protein 1-like (1)	−0.81 (0.196)	−1.84 (0.729)	−0.31 (0.625)	1.22 (0.343)	−0.16 (0.867)	0.83 (0.477)	−0.84 (0.256)	−2.37 (0.369)	−0.40 (0.662)	−1.64 (0.920)
724312	vanin-like protein 1-like (2)	0.04 (0.665)	−4.64 (0.110)	0.06 (0.885)	−**3.63 (0.029)**	0.04 (0.885)	−**3.72 (0.029)**	−0.46 (0.471)	−4.87 (0.100)	−0.11 (0.885)	−**5.24 (0.029)**
494523	glutathione peroxidase-like 1	0.18 (0.471)	−1.24 (0.885)	−0.11 (0.665)	−0.51 (0.312)	0.13 (0.471)	−0.89 (0.817)	−0.11 (0.471)	−1.75 (0.105)	0.69 (0.665)	−1.48 (0.721)
726269	glutathione peroxidase-like 2	−0.30 (0.381)	−2.14 (0.305)	−0.13 (0.720)	−1.85 (0.224)	0.04 (0.916)	−1.50 (0.264)	−0.04 (0.884)	−1.09 (0.554)	−0.31 (0.488)	−3.33 (0.136)
551044	glucose dehydrogenase 2	−1.29 (0.061)	0.31 (0.194)	−0.39 (0.471)	1.00 (0.194)	−0.93 (0.194)	−0.22 (0.665)	−**2.27 (0.030)**	−0.30 (0.817)	−1.07 (0.112)	−0.29 (0.112)
413247	carboxylesterase clade I, member 1	0.17 (0.885)	−1.65 (0.884)	−0.19 (1)	−0.21 (0.309)	0.27 (0.561)	−1.12 (0.885)	−0.10 (0.772)	−1.70 (0.481)	0.00 (0.885)	−1.63 (0.468)
726134	carboxylesterase	−0.07 (0.784)	−3.07 (0.213)	−0.38 (0.174)	−1.22 (0.589)	0.11 (0.582)	−1.53 (0.344)	−0.29 (0.200)	−3.36 (0.066)	−0.37 (0.438)	−3.34 (0.163)
406122	actin related protein 1	−0.07 (0.885)	−2.15 (0.665)	−0.17 (0.885)	−0.92 (0.885)	−0.69 (0.312)	−1.82 (0.665)	−0.72 (0.471)	−3.31 (0.105)	−0.38 (0.471)	−3.84 (0.312)

Log fold change is given together with the p-value in parentheses. Significant expression changes (p-value ≤0.05) are shown in bold.

Four genes thought to be involved in immunity were downregulated in response to several treatments. The gene encoding the serine protease 40 (Gene ID 409626) was downregulated at day 7 in honeybees infected by *N. ceranae*, whatever they were exposed to one insecticide or not, and at day 11 in individuals exposed to the combination *N. ceranae*-imidacloprid. The lysozyme 1 encoding gene (Gene ID 725110) was downregulated at day 11 in honeybees exposed to fipronil or imidacloprid alone and in honeybees exposed to *N. ceranae*-fipronil. The expression of the antimicrobial peptide hymenoptaecin encoding gene (Gene ID 406142) was significantly inhibited at day 11 in response to *N. ceranae* or fipronil, but not to the *N. ceranae*-fipronil combination. In contrast, it was significantly lower in response to the *N. ceranae*-imidacloprid treatment at the same day. At last, honeybees exposed to the *N. ceranae*-fipronil combination showed a decrease in the expression of the gene encoding the glucose dehydrogenase 2 (Gene ID 551044) at day 7.

Other functional groups seemed affected by the applied treatments. Eleven days after the experiment initiation, honeybees infected by *N. ceranae* showed a strong decrease in the expression of a catalase encoding gene (Gene ID 443552), which might be involved in antioxidant reactions and in xenobiotic detoxification. Two genes related to carbohydrate metabolism were significantly downregulated in our experiment: the trehalase encoding gene (Gene ID 410484) in response to *N. ceranae* at day 11 and to *N. ceranae*-fipronil combination at days 7 and 11, and the GMC oxidoreductase 3 encoding gene (Gene ID 410747) in response to all treatments but imidacloprid alone at day 7 and to *N. ceranae*-imidacloprid combination at day 11. The expression of the transcription factor Lim3 homeobox (Gene ID 410658) was significantly downregulated at day 7 in all experimental groups compared to control, and at day 11 in honeybees infected by *N. ceranae*, whether they were exposed to insecticides or not. Honeybees exposed to fipronil or imidacloprid or *N. ceranae*-imidacloprid showed a significant decrease in the expression of a gene encoding a vanin 1-like protein (Gene ID 724312). Vanin is an enzyme with pantetheinase activity that is suspected to have antiparasitic properties against *Plasmodium* in mice [47]. Finally, the only gene overexpressed in our experiment was the chitinase 5 encoding gene (Gene ID 551600) whose expression was significantly increased at day 7 in honeybees infected by *N. ceranae*.

#### Quantitative RT-PCR and RNA-Seq correlation

Gene expression profiles collected at day 7 from experimental groups exposed to no treatment, *N. ceranae* and fipronil, applied alone or in combination, were compared to RNA-Seq data (Figure S3). A strong correlation was found between Exp. 1 and Exp. 2 (Spearman rank correlation ρ = 0.84, n = 92, p<0.001). Among the six genes showing differential expression in RNA-Seq data and chosen for qPCR analysis, four showed positively correlated expression in Exp. 2, while none showed divergent expression. More precisely, honeybees treated with *N. ceranae* alone or with the *N. ceranae*-fipronil combination showed in both experiments a higher expression of the chitinase 5 encoding gene (Gene ID 551600) and a lower expression of the SP 40 (Gene ID 409626) and Lim3 homeobox (Gene ID 410658) encoding genes. The latter was also repressed by fipronil in the two experiments. Similarly, a significant decrease in the expression of the trehalase encoding gene (Gene ID 410484) was observed in response to the *N. ceranae*-fipronil combination in both experiments. Overall, the similar survival and gene expression profiles obtained from the two distinct experiments, using different analytical methods (RNA-Seq and qPCR) and different fipronil concentrations (1.3 and 2 μg/L), showed that the stresses applied induced a reproductible response.

## Discussion

Newly emerged honeybees were exposed to *N. ceranae*-infection or chronic exposure to an insecticide (fipronil or imidacloprid) or a combination of both parasite and insecticide. It is noteworthy that the provided fipronil and imidacloprid concentrations in our study lie within the range detected in contaminated pollen and nectar stored inside hives [18], [20], [21], [48], [49].

Different impacts of fipronil and *N. ceranae* treatments on honeybee survival were observed in this study compared to previous surveys. Parasite infection had a more severe impact on honeybee survival compared to our previous study where the infection of newly emerged honeybees resulted in a mortality of 39% of individuals 22 days post infection (dpi) [39]. This higher impact was particularly evident in Exp. 2, where the mortality reached a maximum of 51% of honeybees at only 12 dpi (Fig. S2). Moreover, we observed a significant impact on honeybee survival in groups exposed to fipronil concentrations of 1.3 μg/L (Exp. 1) or 2 μg/L (Exp. 2) (Fig. 1 and S2). Therefore the insecticide doses received by honeybees were not sublethal, in contrast to previous studies performed with a comparable chronic exposure to fipronil (1 μg/L) [38], [39]. No synergy between *N. ceranae* and fipronil treatments has been observed in the present work while they led to a synergistic effect on honeybee mortality in previous studies [38], [39]. The absence of synergy could be linked to the already high impact of individual treatments, that could have prevented further potentialization, and all these effects could be due to life-history traits of the sampled honeybee colonies such as resources, other contaminants and pathogens that might have an impact on the honeybee lifespan. Indeed, a contamination by *Varroa destructor* virus sequences was detected in RNA-Seq data and two others viruses, the black queen cell virus and the deformed-wing virus, were also detected by RT-PCR in RNA samples extracted from Exp. 2 (data not shown). The presence of viruses in honeybees during experiments might be more common than thought as another study has also reported a RNA sample contamination by viruses [50].

Compared to fipronil, exposure to imidaloprid (2 μg/L) did not lead to a significant increase in individual mortality implying that doses absorbed by honeybees could be considered as sublethal (Fig. S2). The *N. ceranae*-imidacloprid combination did not lead to a synergistic effect on honeybee mortality. This result is in accordance with the data from Alaux *et al.*, (2010) [37], where a synergy between *Nosema* parasites and imidacloprid occurred only with high concentration (70 μg/L) but not with 0.7 or 7 μg/L of imidacloprid.

Honeybee midgut response to parasitism and exposure to insecticide was analysed in two independent experiments using RNA-Seq and qRT-PCR for Exp. 1 and 2 respectively. A strong correlation was found between gene expression changes between both experiments (Fig. S3) showing that the midgut transcriptional response to treatments was reproducible. A strong effect of honeybee ageing on gene expression profiles was suspected from the principal component analysis performed on RNA-Seq data (Fig. 2). This analysis also suggested that parasite and insecticide treatments had very low impact on global gene expression one day after experiment initiation while they influenced it at day 7. Moreover, a higher number of genes showed expression changes by qRT-PCR at day 11 compared to day 7 suggesting that parasite and insecticide have a growing impact on gene expression with time. Overall, early gene expression changes might reflect honeybee response to a moderate disruption of midgut homeostasis induced by parasite and/or insecticide treatments. On the next days, the amplification regarding gene expression changes might reflect the honeybees inability to recover from this disruption, resulting in a growing imbalance that may lead to premature death.

A significant downregulation of several genes potentially implicated in the immune response was detected in *N. ceranae*-infected honeybees at days 7 or 11. Those included the genes encoding the serine proteases SP22 and SP40, glucose dehydrogenase 2, lysozyme 1, hymenoptaecin and GMC oxidoreductase 3 (Tables 1 and 2). In addition to their function in digestion of food, serine proteases in insects participate in regulatory cascade reactions linked to immune responses resulting in rapid activation of the Toll and prophenoloxidase pathways [51]. Antimicrobial peptides such as hymenoptaecin are key elements of the insect innate immunity against bacteria and fungi [52]. Hymenoptaecin was the only antimicrobial peptide significantly affected under *N. ceranae*-infection in our study while abaecin, apidaecin and defensin encoding genes were also downregulated in other studies [8], [9]. Glucose dehydrogenases are components of the humoral immune response associated with melanised encapsulation [53] and have already been shown to be repressed in response to *N. ceranae* [8]. A recent survey revealed a possible implication of GMC oxidoreductases in insect immunity. Related genes are upregulated in silkworm in response to four different pathogens and the knockdown of these genes affects the survival rate of infected individuals [54]. Other genes were shown to be downregulated in *N. ceranae*-infected midguts in previous studies [8]–[10], but not significantly in the present one, such as the *basket* and *u-shaped* genes whose orthologs in *Drosophila melanogaster* are related to immunity [10]. Altogether these data suggest an impairment of the honeybee immune defence in response to *N. ceranae* infection that may favour the parasite development.

Pesticides might also act on insects immune system [22], [55] and fungicides and acaricides have been shown to downregulate immune-related genes in honeybees [55]–[57]. In our study, the hymenoptaecin and lysozyme 1 transcript levels were significantly lower following the chronic exposure to fipronil and to fipronil and imidacloprid respectively (Table 2). Therefore phenylpyrazole and neonicotinoid insecticides might also alter the honeybee immune response.

A significant overexpression of a chitinase encoding gene was detected in honeybees exposed to *N. ceranae*, alone or in combination with an insecticide, and two genes encoding cuticular proteins carrying a chitin-binding domain were activated in honeybees exposed to parasite-insecticide treatments. For the first time data suggest that cuticle coatings, which constitute important barrier defence in insects, might undergo significant modifications in response to a parasite infection. One should also consider that the peritrophic matrix surrounding the food bolus and protecting epithelial cells contains chitin [58], therefore infection by *N. ceranae* might lead to an alteration of the peritrophic matrix.

In insect midgut, a localized immune response can be implemented by the production of reactive oxygen species (ROS) which are toxic to pathogens [59], [60]. As ROS can also have cytotoxic effects on host tissues, a balance between the generation and elimination of ROS must be maintained [60]. Antioxidant reactions involve enzymes such as glutathione peroxidases, catalases and glutathione-S-transferases, which are also involved in xenobiotic detoxification and particularly in pesticide metabolism. In our study, the expression of one of the honeybee catalase encoding genes (Gene ID 443552) was significantly reduced in the midgut 11 days following *N. ceranae* infection (Table 2). In contrast, in *N. ceranae*-infected honeybees at 7 dpi, Dussaubat *et al.*, (2012) [10] observed the overexpression of the same catalase encoding gene, as well as other genes encoding a glutathione peroxidase-like 2 and two cytochrome P450 monooxygenases (CYP/P450s). As the honeybee genome carries several homologs of these genes, the impact of *N. ceranae* infection on antioxidant and detoxification processes appears unclear although there seems to be a reorganization between transcripts. The measure of enzyme activities in *N. ceranae*-infected midguts suggested that such gene expression rearrangement would lead to a decrease in glutathione peroxidase and an increase in glutathione-S-transferase activities [10], [38].

In our study, neither insecticide nor parasite-insecticide treatments led to significant changes in the expression of genes related to detoxification. On the contrary, a significant overexpression of 9 genes encoding CYP/P450s was detected in honeybee larvae orally exposed to imidacloprid (2 μg/L) for 15 days [61]. In insects, CYP/P450s are involved in the resistance to insecticides [62]–[66]. Modifications of detoxifying activities were also observed in honeybees after an acute and topical exposure to sublethal doses of the neonicotinoid thiamethoxam [67]. The low expression of these genes, that did not pass the statistical screening test in the present work, could be linked to the high mortality observed in honeybees exposed to fipronil.

Modifications of the expression of several genes related to trehalose metabolism were observed in our study. A trehalase-encoding gene was highly downregulated in *N. ceranae*-infected honeybees at day 11 (Table 2), as expected from previous data [10]. This gene and two other encoding facilitated trehalose transporters were also downregulated in honeybees exposed to the *N. ceranae*-fipronil combination. Trehalases hydrolyse trehalose to generate glucose which can then be catabolised through glycolysis or the pentose phosphate pathway [68]. In the haemolymph of *N. ceranae*-infected foragers, the trehalose amount has been shown to be lower compared to uninfected individuals while glucose amount remained stable, suggesting that parasitism increased honeybee energetic demand [12]. Trehalases are also involved in various other physiological processes in insects such as regulation of chitin biosynthesis, flight metabolism or cold tolerance [69], [70]. Regulation of a trehalase gene may thus be linked to a complex metabolic response but it would need confirmation through trehalase activity monitoring.

Several genes encoding transcription factors were significantly downregulated in response to parasite and/or insecticide treatment (Tables 1 and 2), including three homeobox-containing proteins (namely the homeodomain interacting protein kinase, Lim3 homeobox and homeobox protein Nkx-2.5-like), referred to as Hox proteins. Another Hox gene, encoding an ortholog of the *D. melanogaster* pituitary homeobox homolog 1 was also found significantly downregulated in the midgut of honeybees infected by *N. ceranae* for 7 days [10]. Hox proteins are transcription factors that regulate the expression of genes involved in growth and differentiation during the developmental processes of animals, from flies to mammals [71]. Hox proteins may also play a role in the innate immune response as modulators of NF-κB-dependent transcription and as mediators of phagocytosis of apoptotic cells [72], [73]. One other transcription factor (annotated as POU domain, class 2, transcription factor 3-like) was significantly repressed in response to fipronil. POU domain-containing transcription factors perform varying functions as regulators of house-keeping genes or as developmental coordinators [74]. In *Drosophila*, POU proteins were shown to control, together with other regulators, the constitutive expression of antimicrobial peptide genes, thus promoting a first-line defence against infection [75]. The pleiotropic effects of Hox and POU transcription factors make it difficult to interpret their regulation, as it could be linked either to a host response to the stressors (*e.g.* protection, compensation for damages, prevention of parasite development) or, in the case of infection, to a host manipulation by the parasite.

## Conclusion

**Figure 3:**
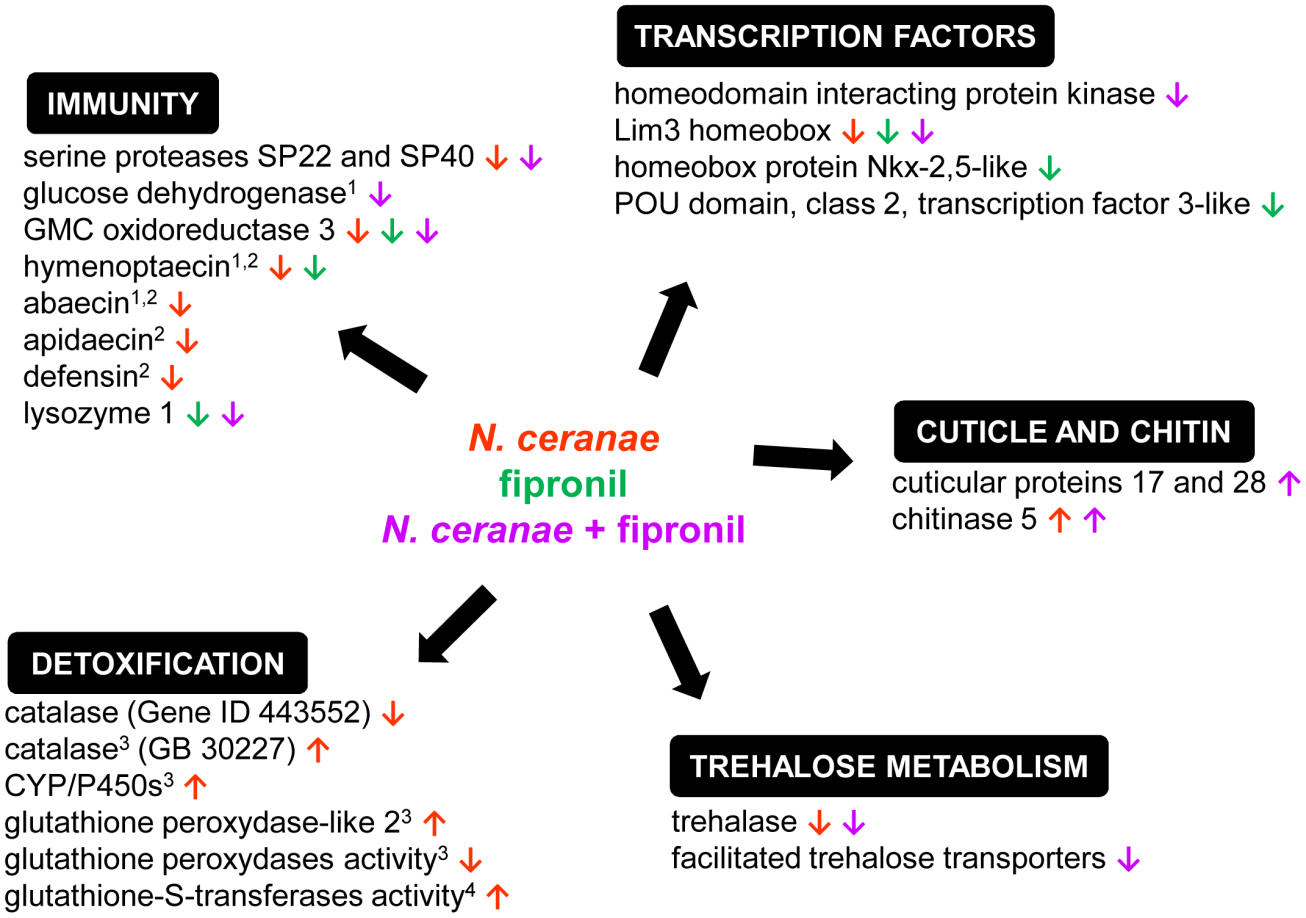
Impact of *N. ceranae* and fipronil on gene expression or enzyme activity in the honeybee. The figure presents a selection of genes or enzymes whose expression or activity has been shown to significantly increase (**↑**) or decrease (**↓**) under exposure to *N. ceranae* (red), to fipronil (green), or to a *N. ceranae*-fipronil combination (purple) in the present work or in previous studies: ^1^Antunez *et al.*, 2009; ^2^Chaimanee *et al.*, 2012; ^3^Dussaubat *et al.*, 2012; ^4^Vidau *et al.*, 2011.

Our result showed that *N. ceranae*-fipronil and *N. ceranae*-imidacloprid combinations do not systematically lead to a synergistic effect on honeybee mortality. Such variability in impact on mortality could be due to additional unexpected stressors related to life-history traits of the sampled honeybee colonies. In our study, gene expression profiles in honeybee midgut showed that insecticide treatments had no impact on detoxifying genes but led to a significant downregulation of immunity-related genes (Figure 3), suggesting a possible immunotoxicity of neonicotinoid and phenylpyrazole insecticides under chronic exposure. Honeybees treated with *N. ceranae*, alone or in combination with an insecticide, showed a strong alteration of midgut immunity visible after 7 days, together with significant modifications affecting barrier defence and trehalose metabolism. The increasing impact of the treatments with time suggests a growing imbalance of the honeybee transcriptome that would reflect an absence of stress recovery and could explain the observed higher mortality rates.

## Supporting Information

Figure S1 **Quantitative RT-PCR validation of RNA-Seq data on a selection of eight genes.** Data show values of differential expression of eight selected genes (*i.e.* encoding chitinase 5, SP14, SP40, Lim3 homeobox, glucosinolate sulphatase, trehalase, hydrocephalus-inducing protein-like and actin related protein 1) for the same pairwise comparisons between experimental groups, determined by RNA-Seq and qPCR. A strong correlation was found between qRT-PCR and RNA-Seq data (Spearman rank correlation p = 0.722, n = 72, p<0.001).
(TIF)

Figure S2 **Effect of** ***N. ceranae*** **and insecticide, acting alone or in combination, on honeybee survival.** Data give the cumulative proportion of surviving honeybees exposed to no treatment (blue), *N. ceranae* (red), insecticide (green), or a *N. ceranae*-insecticide combination (pink). *N. ceranae*-treated honeybees were individually infected at their emergence (day 0) and insecticide-treated ones were chronically and orally exposed to **(A)** fipronil (2 μg/L) or **(B)** imidacloprid (2 μg/L) from day 0 to day 7. Data from 140 honeybees per experimental condition were analysed with the Kaplan-Meier method.
(TIF)

Figure S3 **Comparison between transcripts abundance of the same set of 24 genes determined in Exp. 1 (RNA-Seq) and Exp. 2 (qRT-PCR) at day 7.** The log_2_ of mean between replicates transcripts counts is given for each gene and each experimental group (*i.e.* control, *N. ceranae*, fipronil, *N. ceranae*-fipronil). Ct determined by qRT-PCR in Exp. 2 was normalized using gene RpS5a as the reference. A strong correlation was found between Exp. 1 and Exp. 2 (Spearman rank correlation p = 0.84, n = 92, p<0.001).
(TIF)

Table S1 **List of primer sequences and conditions used for quantitative RT-PCR analysis in this study.** Nucleotide sequences for both forward (F) and reverse (R) primers are given together with the amplicon size, the primer final concentration and hybridization temperature used for amplification, as well as the linearity and the efficiency of the qPCR.
(DOCX)

Table S2 **Honeybee genes detected in honeybees exposed to no treatment (Ct),** ***N. ceranae*** **(Ncer), fipronil (Fip), or a** ***N. ceranae*** **-fipronil (Nc+F) at day 1 (D1) or 7 (D7).** The table gives the normalized counts for each sample together with the mean log_2_ of replicates for each condition and the log_2_ fold changes (and adjusted p-value in parenthesis) for each pairwise comparison.
(XLSX)
