# Supplementary figures and images for: Transcriptome Analyses of the Honeybee Response to Nosema ceranae and Insecticides
Source: PLoS One. 2014 Mar 19;9(3):e91686. doi: 10.1371/journal.pone.0091686 (PMC3960157; doi:10.1371/journal.pone.0091686)

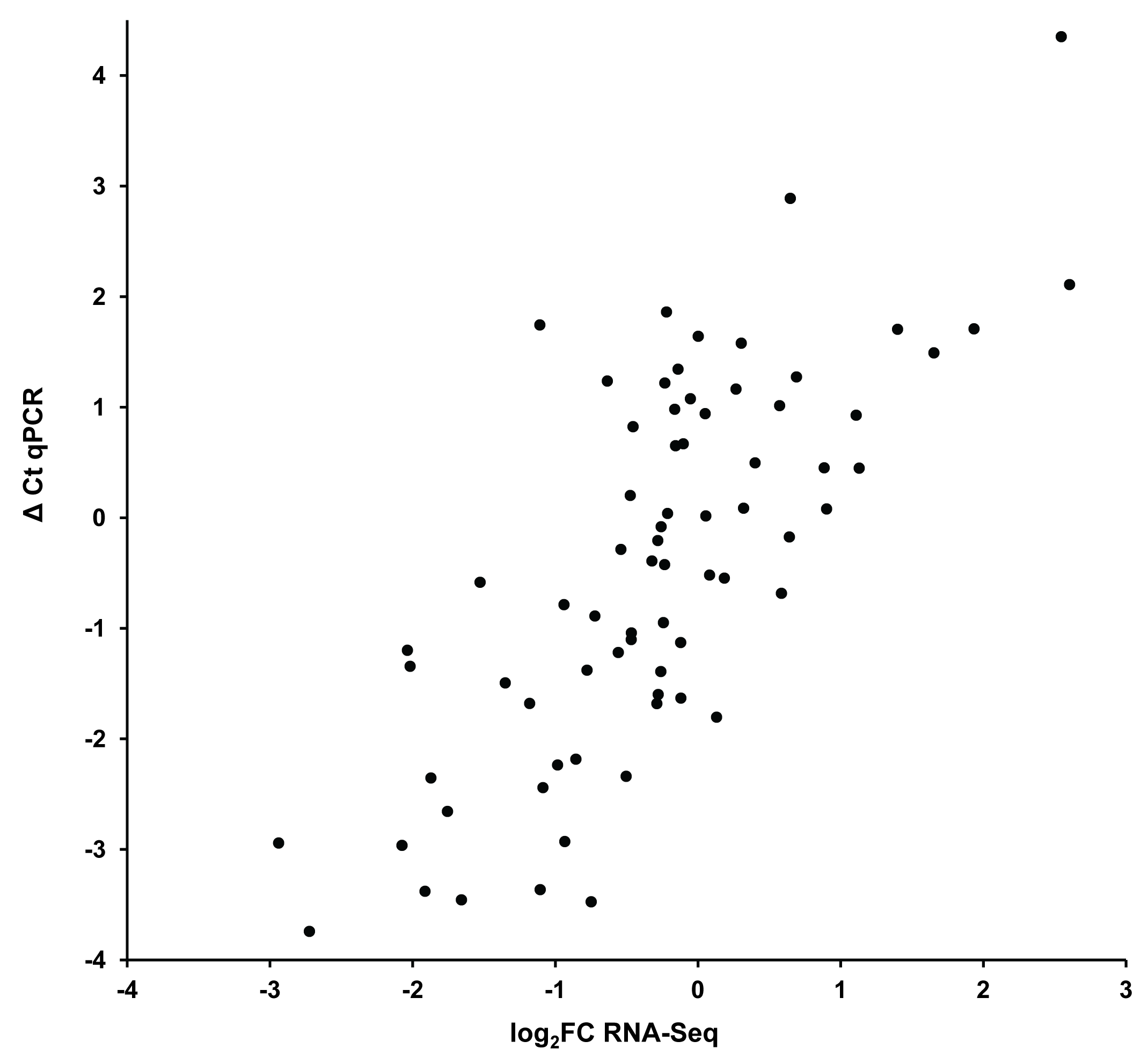

Supplement: Figure S1 — Quantitative RT-PCR validation of RNA-Seq data on a selection of eight genes. Data show values of differential expression of eight selected genes (i.e. encoding chitinase 5, SP14, SP40, Lim3 homeobox, glucosinolate sulphatase, trehalase, hydrocephalus-inducing protein-like and actin related protein 1) for the same pairwise comparisons between experimental groups, determined by RNA-Seq and qPCR. A strong correlation was found between qRT-PCR and RNA-Seq data (Spearman rank correlation p = 0.722, n = 72, p<0.001). (TIF) [file pone.0091686.s001.tif]

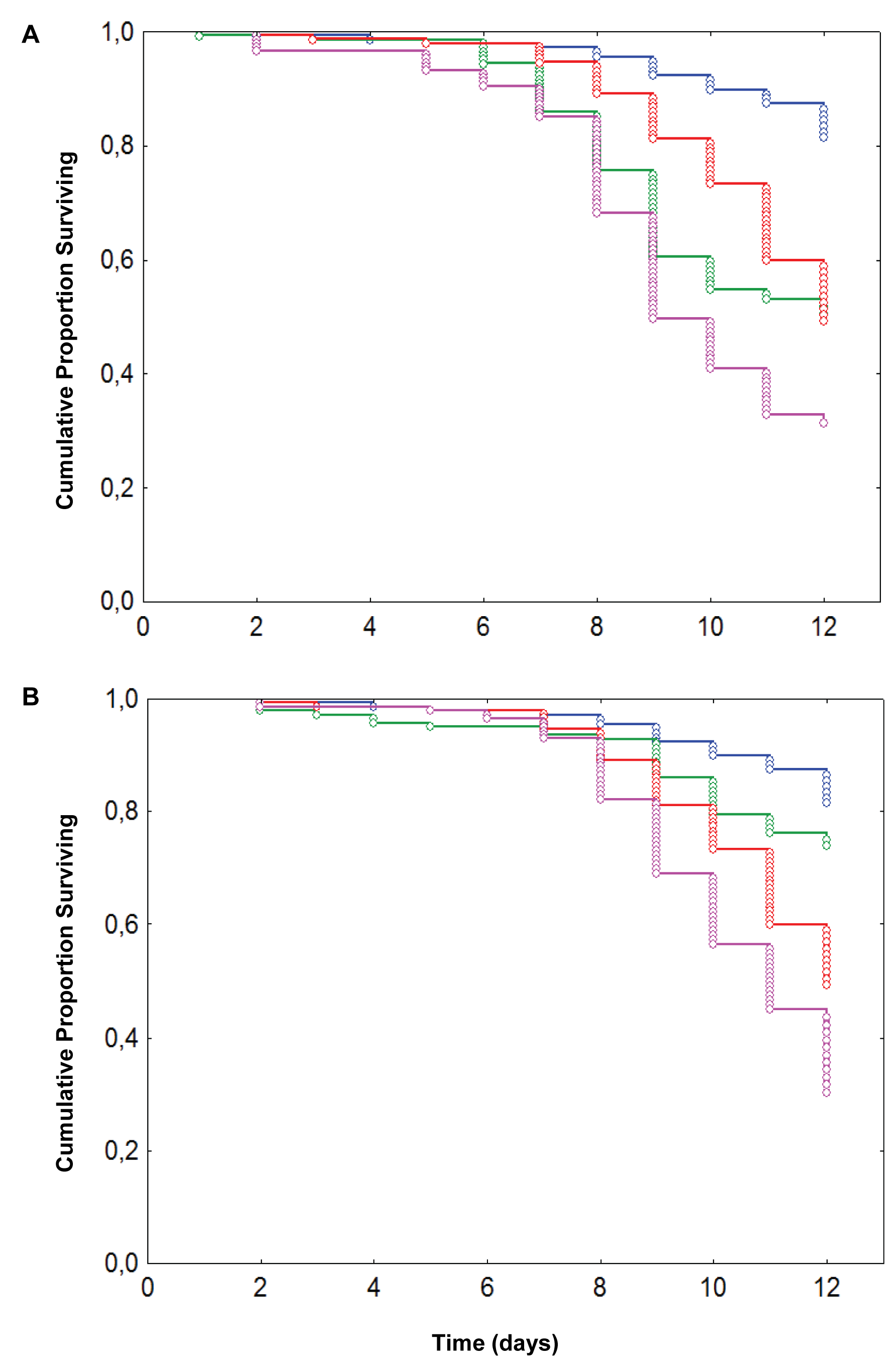

Supplement: Figure S2 — Effect of N. ceranae and insecticide, acting alone or in combination, on honeybee survival. Data give the cumulative proportion of surviving honeybees exposed to no treatment (blue), N. ceranae (red), insecticide (green), or a N. ceranae-insecticide combination (pink). N. ceranae-treated honeybees were individually infected at their emergence (day 0) and insecticide-treated ones were chronically and orally exposed to (A) fipronil (2 μg/L) or (B) imidacloprid (2 μg/L) from day 0 to day 7. Data from 140 honeybees per experimental condition were analysed with the Kaplan-Meier method. (TIF) [file pone.0091686.s002.tif]

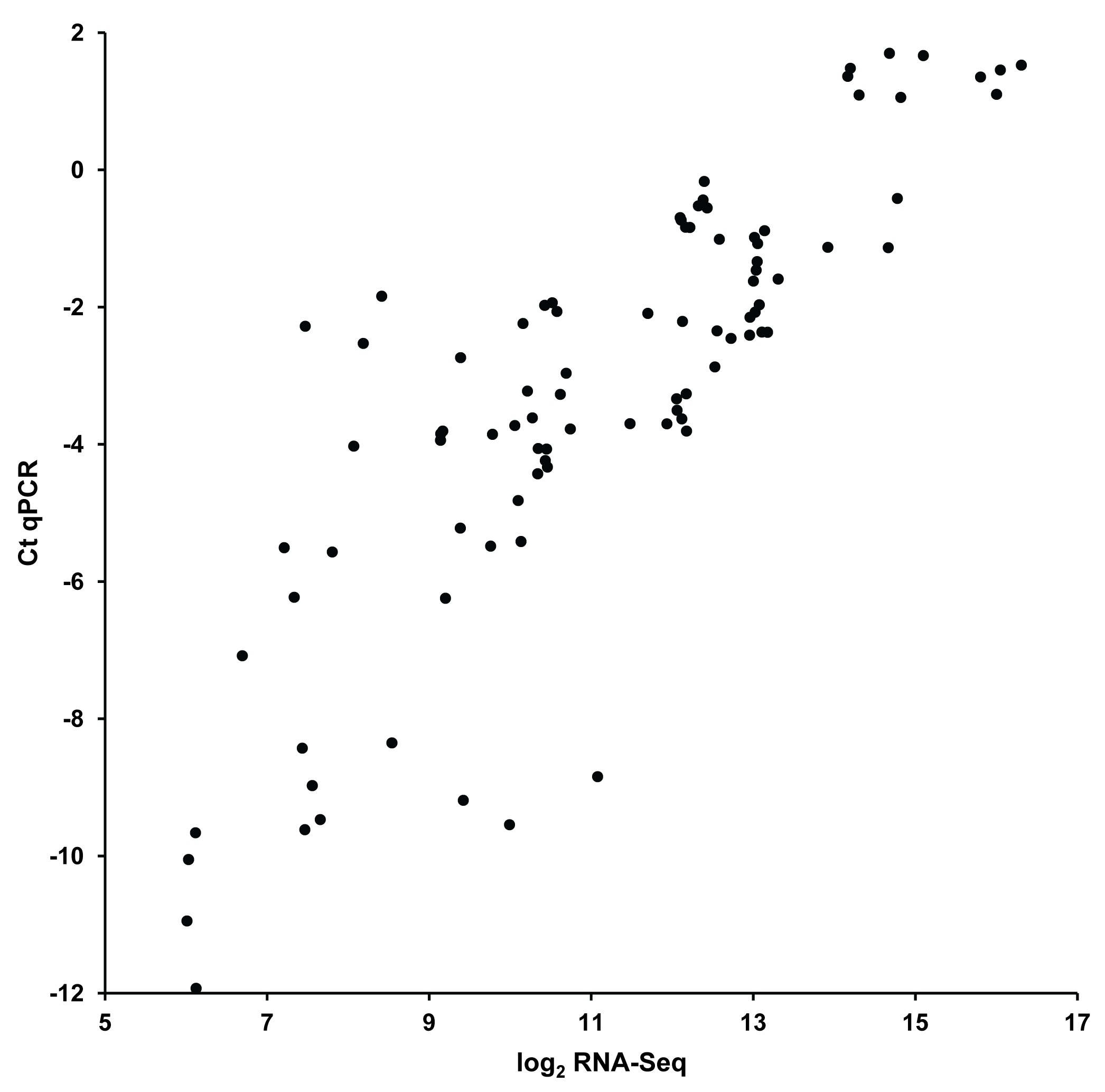

Supplement: Figure S3 — Comparison between transcripts abundance of the same set of 24 genes determined in Exp. 1 (RNA-Seq) and Exp. 2 (qRT-PCR) at day 7. The log2 of mean between replicates transcripts counts is given for each gene and each experimental group (i.e. control, N. ceranae, fipronil, N. ceranae-fipronil). Ct determined by qRT-PCR in Exp. 2 was normalized using gene RpS5a as the reference. A strong correlation was found between Exp. 1 and Exp. 2 (Spearman rank correlation p = 0.84, n = 92, p<0.001). (TIF) [file pone.0091686.s003.tif]
