# Supplementary material for: Transcriptome Analyses of the Honeybee Response to Nosema ceranae and Insecticides
Source: PLoS One. 2014 Mar 19;9(3):e91686. doi: 10.1371/journal.pone.0091686 (PMC3960157; doi:10.1371/journal.pone.0091686)
Supplement: Table S1 — List of primer sequences and conditions used for quantitative RT-PCR analysis in this study. Nucleotide sequences for both forward (F) and reverse (R) primers are given together with the amplicon size, the primer final concentration and hybridization temperature used for amplification, as well as the linearity and the efficiency of the qPCR. (DOCX) [file pone.0091686.s004.docx]

| Product | Gene (locus) | Primer sequences | Reference | Size (pb) | Conc. (nM) | T hyb (°C) | Linearity (r²) | Efficiency (%) |
| --- | --- | --- | --- | --- | --- | --- | --- | --- |
|  |  |  |  |  |  |  |  |  |
| actin related protein 1 | 406122 | β-actin-F : ATGCCAACACTGTCCTTTCTGG  β-actin-R : GACCCACCAATCCATACGGA | Yang and Cox-Foster, 2005 | 151 | 300 | 56 | 0.998 | 0.90 |
| carboxylesterase | 726134 | CaE2-F2 : CGACGAGGTAGCAGAGAATCC  CaE2-R2 : ATTATGACCGGGACATCGGC | this work | 81 | 300 | 58 | 0.998 | 0.93 |
| carboxylesterase clade I, member 1 | 413247 | CaE1-F : TGTGTCATTGGTCCAGAGCC  CaE1-R : ACACCTTGATCCTTGCTCGG | this work | 117 | 300 | 58 | 0.999 | 0.91 |
| catalase (1) | 411758 | CAT1-F2 : CATTTCCAGATGGCGTTGGC  CAT1-R2 : TGCACTTCCAGCATCACCAA | this work | 80 | 300 | 58 | 0.999 | 0.98 |
| catalase (2) | 443552 | CAT2-F : AAGCCAGTTTGACGGTAGGG  CAT2-R : AGCATGGACTACACGTTCCG | this work | 113 | 300 | 58 | 0.999 | 0.92 |
| chitinase 5 | 551600 | Endochiti-F : TATCGAAAGGACGTCGGCAG  Endochiti-R : TCACGCCGATGAACGAGTAG | this work | 85 | 300 | 58 | 0.998 | 0.99 |
| defensin 1 | 406143 | Defensin-F1 : TGCGCTGCTAACTGTCTCAG  Defensin-R1 : CGTTTCGGTTAAGTGCCATT | Evans, 2006 | 119 | 400 | 54 | 0.999 | 0.97 |
| endoplasmin-like | 412150 | HSP90-F : TTCTCCATGTGCTTTGGTTG  HSP90-R : TTTTTCTGGGGATCATCAGTC | this work | 102 | 300 | 55 | 0.999 | 1.05 |
| glucose dehydrogenase 2 | 551044 | GLD3-F : GAACGAGAAAAACGCCTGTC  GLD3-R : CCCAATCGTCGTAATCCTTG | this work | 131 | 300 | 57 | 0.997 | 1.01 |
| glucosinolate sulphatase | 551758 | GLSS-F2 : ATTGGAGAAAGGAGCCCGTG  GLSS-R2 : CTTCAGATCTCCACCAGCCG | this work | 91 | 300 | 58 | 0.999 | 1.01 |
| glutathione peroxidase-like 1 | 494523 | Gtpx1-F2 : AGATTCTGCGCATCCTTTATGG  Gtpx1-R2 : CGTTCCACCACTTTGCCTTC | this work | 120 | 300 | 58 | 0.999 | 0.91 |
| glutathione peroxidase-like 2 | 726269 | Gtpx2-F : GGAAGTGGTTGAAGACACAAGC  Gtpx2-R : ACAGTTGGTGCAAATCGAGA | this work | 115 | 300 | 58 | 0.999 | 0.92 |
| glutathione S-transferase S1 | 552304 | GSTS1-F : ATTCCTGGTCTTGCCGAACC  GSTS1-R : TGGGCCATTCTTCGATATCAATTC | this work | 91 | 300 | 56 | 0.999 | 0.94 |
| GMC oxidoreductase 3 | 410747 | GlucDSH-F2 : GATGGTCGCGGAAAAAGCAG  GlucDSH-R2 : AACAGCGCCCTCTACATTCC | this work | 96 | 300 | 58 | 0.999 | 0.94 |
| hydrocephalus-inducing protein-like | 100578512 | HydIP-F : ATTTGTCGAACGTGGAGACG  HydIP-R : GGCGCCAAGCACAAATTCAC | this work | 91 | 300 | 58 | 0.998 | 1.05 |
| hymenoptaecin | 406142 | Hymenoptaecin-F : CTCTTCTGTGCCGTTGCATA  Hymenoptaecin-R : GCGTCTCCTGTCATTCCATT | Evans, 2006 | 200 | 400 | 54 | 0.996 | 1.05 |
| Lim3 homeobox | 410658 | Lim3-F : AGAGTCTCAAAACGTCCGGC  Lim3-R : GATCCAGAGGGTGCATCTCG | this work | 101 | 300 | 58 | 0.998 | 0.95 |
| lysozyme 1 | 725110 | LYS-F : ACACGGTTGGTCACTGGTCC  LYS-R : GTCCCACGCTTTGAATCCCT | Yang and Cox-Foster, 2005 | 201 | 300 | 60 | 0.998 | 0.94 |
| POU domain, class 2, transcription factor 3-like | 727092 | POU-F2 : TCGTGCAAAATCCAAAGCCC  POU-R2 : ACCACACTCGAACGACTTCC | this work | 87 | 300 | 58 | 0.999 | 1.02 |
| ribosomal protein S5a | 409728 | RPS5-F : AATTATTTGGTCGCTGGAATTG  RPS5-R : TAACGTCCAGCAGAATGTGGTA | Evans, 2006 | 115 | 300 | 56 | 0.997 | 0.86 |
| serine protease 14 | 725154 | SP14-F : CGAAAAACACTACGTGGGGC  SP14-R : TGTCTCCTTGACAAGCGTCC | this work | 110 | 300 | 58 | 0.997 | 1.01 |
| serine protease 40 | 409626 | SP40-F : TTTCCTCCGTCCCTCTTCCA  SP40-R : CTAAGTCTGCCCCAGCCAG | this work | 82 | 300 | 58 | 0.999 | 0.97 |
| trehalase | 410484 | Trehalase-F2 : GTTCGGATGGACTAACGGGG  Trehalase-R2 : CGTCCTCGTATCCAACCTCC | this work | 83 | 300 | 58 | 0.999 | 1.02 |
| vanin-like protein 1-like (1) | 100578995 | Vanin1-F2 : ACGTTTGATCTTCATAACAAACCAG  Vanin1-R2 : TGGTCATCGAATCATTGAGCAG | this work | 109 | 300 | 58 | 0.999 | 0.96 |
| vanin-like protein 1-like (2) | 724312 | Vanin2-F2 : CGAAGGAGCGGCTTTTAGTC  Vanin2-R2 : TCGATGTCCGATGTATGCAG | this work | 118 | 300 | 56 | 0.998 | 0.97 |
